# Testing the informal care model: intrapersonal change in care provision intensity during the first lockdown of the COVID-19 pandemic

**DOI:** 10.1007/s10433-022-00713-2

**Published:** 2022-06-22

**Authors:** Klara Raiber, Ellen Verbakel, Alice de Boer

**Affiliations:** 1 Radboud University in Nijmegen, P.O. Box 9104, 6500 HE Nijmegen, The Netherlands; 2 The Netherlands Institute for Social Research, The Hague, The Netherlands; 3 Free University of Amsterdam, Amsterdam, The Netherlands

**Keywords:** Informal care model, Intensity, Theory testing, COVID-19

## Abstract

Informal care, meaning taking health-related care of people in their own social network, is a topic that gets more and more attention in social science research because the pressure on people to provide informal care is rising due to ageing societies and policy changes. The *Informal Care Model* developed by Broese van Groenou and de Boer (Eur J Ageing 13(3):271–279, 2016) provides a theoretical foundation to understand under what conditions a person provides informal care. We test this theoretical model by applying it to intrapersonal changes in informal care provision during the first COVID-19 lockdown in the Netherlands in Spring 2020. Data from the LISS panel from two time points, March 2020 and data from July over the period of April/May 2020, were analysed with multinominal multilevel regression analysis (*N* = 1270 care situations of 1014 caregivers). Our results showed that the individual determinants (*Do I have to?, Do I want to?,* and especially *Can I?*) discussed in the Informal Care Model (apart from a series of control variables) are contributing substantially to the understanding of intrapersonal changes in care provision during the first lockdown and by that, we found empirical support for the theoretical model. We conclude that on top of its original purpose to explain between-individual differences in informal caregiving using static indicators, the *Informal Care Model* is also useful to explain intrapersonal changes in informal caregiving using dynamic indicators.

## Introduction

The need for informal care, meaning health-related care of older, disabled, or sick people in the social network (thus excluding regular childcare, care provided as part of one’s occupation or as a volunteer), is expected to increase, mainly because of two reasons. First, with an ageing society more and more older people have complex health issues and need care (Kromhout et al. 2018). Second, in many countries, including the Netherlands, more responsibility is put onto families and it is expected that people in the personal network provide care (Broese van Groenou et al. 2016). However, there might not be enough informal caregivers available considering that families are getting smaller, the labour market participation, especially of women and older workers, is increasing (Agree and Glaser 2009; Broese van Groenou and de Boer 2016; Robine et al. 2007). Greater labour force participation, especially among older workers and women, is even needed to offset the rising costs of welfare state provisions to the ageing population (Plaisier et al. 2015).

The recent COVID-19 pandemic and the related measures taken by governments can be seen as a unique historical setting to evaluate factors influencing individual informal care provision. So far, scholars have mainly focused on the consequences of the pandemic on informal care (Bergmann and Wagner 2021; Carers UK 2020; Chan et al. 2020; Evandrouet al. 2020; Giebel et al. 2021; Gräler et al. 2020; Lorenz-Dant and Comas-Herrera 2021; Raiber and Verbakel 2021; Rodrigues et al. 2021). Less is, however, known about the determinants of the informal care provision and how the social context influences the starting point of informal care provision. For this, the *Informal Care Model* by Broese van Groenou and de Boer (2016) provides a theoretical foundation to better understand and analyse care provision. It combines individual reasons for care provision with how the social context can facilitate or hinder care provision. In brief, it distinguishes factors related to *Can I*, *Do I have to?*, and *Do I want to?* provide care. Many scholars directly built their work on the model (for instance, Brettschneider et al. 2019; Calvó-Perxas et al. 2018; de Jong et al. 2021; Kaschowitz & Brandt 2017; Palmer 2019; Peng and Anstey 2019; Suanet et al. 2019; Verbakel, et al. 2017; Vos et al. 2020). Directly empirical assessing the *Informal Care Model*, de Klerk et al. (2021) identified, in line with the *Informal Care Model*, both sociodemographic factors as well as individual barriers and beliefs as determinants for different types of informal care relationships in the Netherlands. Tur-Sinai et al. (2019) also validated the model and found that social context and barriers play an important role in the informal care provision in Italy and Israel.

What is currently missing is an intrapersonal assessment of the *Informal Care Model*, including dynamic indicators of *Can I*, *Do I have to?*, and *Do I want to?* influencing changes in the intensity of caregiving. If the *Informal Care Model* appears also useful for explaining intrapersonal changes in informal caregiving, it will not only help us to gain better insight into mechanisms behind changes in informal care provision; it will also provide us with clues on how to meet the increasing demand for informal care in the near future. We aim to fill this gap and test the model for a very specific setting, namely the COVID-19 pandemic as an external shock onto the informal care provision.

We add to the literature by using the external shock of the pandemic as a form to evaluate the scope of an existing theoretical model by testing it from a dynamic rather than static perspective. We assess with quantitative data how the restrictions of the Spring 2020 lockdown changed the intensity of care provision of Dutch caregivers who were already providing care before the lockdown. Thereby we test the relationship between interpersonal change in care provision and the three individual determinants of the *Informal Care Model*: (1) perceived constraints (*Can I?*) (2) normative beliefs (*Do I have to?*), and (3) general beliefs (*Do I want to?*). We base our analysis on an assembled survey study of the Dutch Longitudinal Internet studies for the Social Sciences (LISS). Caregivers who indicated that they provided informal care in March 2020 were asked follow-up questions in July 2020 about how the COVID-19 lockdown affected the intensity of their informal caregiving provision. We run multilevel multinomial logistical regression (*N* = 1270 informal care situations of 1014 informal caregivers).

## The *informal care model*

Building on an extensive literature review as well as other theoretical models, Broese van Groenou and de Boer (2016) developed a model—the *Informal Care Model*—that gives the theoretical foundation to explain why an individual provides care when being in the situation where a person in their personal network needs help. The model looks at the individual dispositions of the caregiver but at the same time puts it in a wide framework including the context like the family and social network, or community care and technology. Further, the model is embedded in macro-level structures like the ageing population and long-term care policies, the labour market, and socio-cultural norms (Broese van Groenou & de Boer 2016). In this article, we focus on the individual determinants and test if the assumptions made by Broese van Groenou and de Boer (2016) hold when applying it to an external shock, the COVID-19 pandemic. In the following, we will describe each of the three individual determinants separately and relate it to the specific situation during the Spring 2020 lockdown in the Netherlands.

The first determinant is about general beliefs and the question of whether one wants to help (*Do I want to?*). Some people have different moral ideas or want to help more than others. The reasons for that vary from a feeling of greater responsibility, over religious reasons, to differences in role expectations (Broese van Groenou and de Boer 2016). The COVID-19 pandemic, being a very uncertain time for many, could have given some people a greater urge to help others. This could be especially true for caregivers who had the feeling that their loved ones had a hard time and needed (more) support (Evans et al. 2020). We, therefore, expect that caregivers who worried more about their care recipients than before the lockdown did increase their care provision.

The second determinant is about the normative belief that one has to care for someone (*Do I have to?*) relating to the social norms of solidarity and reciprocity, meaning that people want to help others because they got help before by the same person or by someone else. The societal context here is the availability of alternative care (Broese van Groenou and de Boer 2016). Caregivers have to do less themselves if formal caregivers or other informal caregivers take over tasks. In times of the first COVID-19 lockdown, the social conditions changed because a lot of formal care to care recipients living at home was scaled back. Care facilities like day-care were closed, and formal home care was less available (Ministry of Health 2020). For reasons of preventing potential infection by the virus, some care receivers or their caregivers might have even decided to reduce formal care willingly (de Vries and Pols 2020). As a result, informal caregivers had to take over tasks previously covered by formal care (De Boer et al. 2020). In summary, we expect that caregivers who faced a reduction in formal care provided to ‘their’ care recipient took over more care and increased their care provision.

Last, the third determinant is about being able to care for someone (*Can I?*) and to overcome perceived barriers like geographic distance, time, money, and competence (Broese van Groenou and de Boer 2016). The restrictions by the Dutch government to prevent the coronavirus to spread can be seen as a new barrier keeping caregivers from providing the same amount of care. Caregivers were less able to care because the restrictions involved (1) the rule to keeping 1.5-m distance making physical contact, and therefore, many care tasks difficult, (2) discouraging social contact, especially to old and sick people, (3) the rule to stay at home with mild symptoms (Government of the Netherlands 2020a), and (4) not being able to visit nursing homes (Government of the Netherlands 2020b). Not only the official lockdown rules may have influenced the restrictions the caregiver felt, but also restrictions put in place by families to limit the risk of transmission to their loved ones with health issues. Further, the lockdown influenced the time availability of caregivers. Some caregivers had increased time pressure, especially informal caregivers with young children (due to the closing of schools and child care facilities) or caregivers with so-called essential jobs that required more time (Yerkes et al. 2020). Other caregivers had more time available than before because work was reduced (or lost), social activities were cancelled, and time was saved by not commuting to work (Giurge et al. 2021). All together, we expect that caregivers who felt more restricted by the new rules of the government were less able to provide care and thus, reduced their caregiving intensity. This also means that caregivers who felt less restricted were more able to provide care and increased their caregiving intensity.

## Methods

### Data

We use quantitative informal caregiving data collected in the Netherlands of two assembled survey studies, thus two time points, of the LISS panel. The LISS panel is administered by CentERdata (Tilburg University, The Netherlands) and has been based on a representative sample of the Dutch population (see www.lissdata.nl and Scherpenzeel and Das 2010). Each month, a core module (which is repeated yearly) is fielded in the LISS panel, complemented with so-called assembled studies: (often one-time only) modules, either to the whole LISS sample or a subsample. We used two assembled studies. The first part of the data we used was collected in March 2020^1^ and was aimed at respondents aged 16–78 who indicated in January 2020 that they cared for someone at least once in their life or at the moment of the interview (name study: ‘Retrospective informal care career: Main measurement’, response rate 84.5%, Verbakel and CentErdata 2021). In the March data, respondents could list a maximum of seven people (care situations) they cared for due to health issues or old age before the moment of the interview or ongoing at the moment of the interview.^2^ For a maximum of three, randomly selected, caregiving situations detailed questions were asked about the informal caregiving situation and caregiver experiences. In case a respondent mentioned more than three situations, a less extensive questionnaire was used for the remainder of care situations. This less extensive questionnaire did not include information that was relevant for this study. We, therefore, excluded the caregiving situations with insufficient information (251 caregiving situations excluded). Caregivers who indicated in March that they were at the moment of the interview providing care were asked in the second assembled study in July 2020 how their caregiving intensity changed due to the COVID-19 pandemic for the period April and May 2020 (name study: ‘Retrospective informal care career: Follow-Up measurement’, response rate 88.7%, Verbakel and CentErdata 2021). We selected caregivers who were still providing care after March or stopped caregiving due to reasons related to the pandemic. We did not include caregiving situations where the care recipient died due to the virus. After listwise deletion (131 cases were excluded), the final sample was including 1270 caregiving situations for 1014 caregivers.

### Measures

*Dependent variable.* Our dependent variable was the change in caregiving intensity separately for each caregiving situation measured by the questions: ‘Did the corona crisis affect the amount of time you helped <  < name care recipient >  > in April/May? Indicate which statement best describes your situation’. The respondents were able to select from five outcomes; cared much less, cared less, continued to care the same amount, cared more, or cared much more. Together with a question of whether the respondent stopped care due to the reasons related to the virus, we built four outcome categories; (1) stopped caregiving, (2) cared less, (3) cared the same amount, and (4) cared more.

*Main predictors.* The theoretical constructs *Do I want to?, Do I have to?,* and *Can I?* were similarly based on questions in the follow-up questionnaire. To measure *Do I want to?* respondents indicated for each caregiving situation whether the statement ‘I was worried about <  < name care recipient >  > ’ was much more, more, equal, less, or much less true during the lockdown, i.e. April/May 2020, than before the lockdown. A high value on this variable was coded as being more worried in April/May than before the lockdown, thus more strongly wanting to help.

*Do I have to?* is strongly related to the reduction in formal care and therefore should capture if there was a change in intensity of formal care. The respondents indicated for each type of formal care (home care, day-care or overnight care, care or nursing home, case manager or residential counsellor) whether that type of formal care was reduced or increased during April/May (or whether the care recipient received the particular type of formal care neither before nor during the lockdown). From that we created dummies indicating if there was (1) no change in formal care (if all types of formal care were stable), (2) formal care was reduced ( if at least one of the formal care types was reduced and none increased), (3) formal care increased (if at least one of the formal care types was increased and none reduced), or (4) formal care was reduced and increased (if some types of formal care were increased but others were reduced).

*Can I?* was measured as the felt restrictions by the rules of the Dutch government with a 5-point-Likert scale (from strongly agree to strongly disagree) on the statement ‘The government's measures to control the coronavirus make it difficult to give aid to <  < name care recipient >  > ’. A higher value stood for not feeling restricted and indicated that the caregiver was more able to provide care.

*Control variables* As control variables, we included variables both on the care situation level and at the caregiver level. These variables are known to be related to caregiving intensity, and we assume that they may also influence changes in intensity. Further, we assume that also *Informal Care Model* determinants *Do I want to?, Do I have to?,* and *Can I?* are potentially influenced by the control variables as they might change the willingness to care, the need or use of formal care, and the perceptions of restrictions (see for example Broese van Groenou et al. 2013). On the caregiving situation level, the social relationship of the caregiver with the care recipient was added with the following categories: (1) partner, (2) (in-law, step-) parent, (3) (step-)child, (4) other family members, or (5) friends, neighbours, acquaintances, or colleagues. Next, the living situation was included with the following categories (1) caregiver and care recipient live in the same household, (2) care recipient lives independently (alone or with someone else), or (3) the care recipient lives in a nursing home or other care institutions. At the same time, we controlled for a range of health indicators of the care recipients of our respondents as recorded in the March data. We included an indicator for memory problems including the categories (1) no memory problems, (2) some memory problems, or (3) serious memory problems. Similarly, mental health problems were controlled for with the categories (1) no mental health problems, (2) some mental health problems, or (3) serious mental health problems. Further, we included sum scores of activities the caregiver needed help with. First, activities of daily living (ADL) with up to three possible activities; walking, dressing, and/or eating. Second, instrumental activities of daily living (IADL) with a sum of up to three possible activities needed help with; housework, shopping, and/or preparing a meal. To control for a potential ceiling effect, meaning that some caregivers might not be able to increase caregiving more, we added the hours spent on caregiving as reported in the pre-measurement in March.

As control variables, we included variables both on the care situation level and at the caregiver level. These variables are known to be related to caregiving intensity, and we assume that they may also influence changes in intensity. Further, we assume that also *Informal Care Model* determinants *Do I want to?, Do I have to?,* and *Can I?* are potentially influenced by the control variables as they might change the willingness to care, the need or use of formal care, and the perceptions of restrictions (see for example Broese van Groenou et al. 2013). On the caregiving situation level, the social relationship of the caregiver with the care recipient was added with the following categories: (1) partner, (2) (in-law, step-) parent, (3) (step-)child, (4) other family members, or (5) friends, neighbours, acquaintances, or colleagues. Next, the living situation was included with the following categories (1) caregiver and care recipient live in the same household, (2) care recipient lives independently (alone or with someone else), or (3) the care recipient lives in a nursing home or other care institutions. At the same time, we controlled for a range of health indicators of the care recipients of our respondents as recorded in the March data. We included an indicator for memory problems including the categories (1) no memory problems, (2) some memory problems, or (3) serious memory problems. Similarly, mental health problems were controlled for with the categories (1) no mental health problems, (2) some mental health problems, or (3) serious mental health problems. Further, we included sum scores of activities the caregiver needed help with. First, activities of daily living (ADL) with up to three possible activities; walking, dressing, and/or eating. Second, instrumental activities of daily living (IADL) with a sum of up to three possible activities needed help with; housework, shopping, and/or preparing a meal. To control for a potential ceiling effect, meaning that some caregivers might not be able to increase caregiving more, we added the hours spent on caregiving as reported in the pre-measurement in March.

On the caregiver level, we controlled for the age of the caregiver, for how many people the caregivers cared for at the moment of the interview, whether or not the caregiver was working in May, whether or not children were living in the household in May,^3^ and the sex of the respondent (men or women).

### Analytical strategy

We run multilevel multinominal logistic regression in STATA 16 (gsem) to account for the structure of the data where caregiving situations are nested in caregivers. We interpreted the effect sizes based on marginal effects at the mean (MEM) meaning predictions of the variable of interest with all other variables kept constant on their average (Mood 2010).

We performed two additional analyses to explore the robustness of our results—and hence, the applicability of the *Informal Care Model*—if we apply stricter definitions of informal care. First, we excluded care recipients who lived in a nursing home or other care institution. Our rationale is that the *Informal Care Model* was originally designed for informal caregivers of community-dwelling care recipients not including caregiving to people in care institutions (Broese van Groenou and De Boer 2016), and caregivers to people in care institutions differ from other caregivers. Previous research showed that these caregivers give less intense help than caregivers of independent living persons, but assist older residents with more types of activities and with more complex care needs (Broese van Groenou 2010). In addition, informal care in institutional settings was likely most rigorously affected by the rules of the government because of the visiting ban in nursing homes (Jeanneau et al. 2020).

Second, we excluded care situations where the only caregiving task before the lockdown was emotional support (14.02%). In other studies, this group often falls outside the definition of informal caregivers. Applied to the COVID 19 pandemic, we think that in such care situations alternatives like (video-)calling were relatively easy to adopt and were thus less influenced by the lockdown measures than care situations that (also) involved practical or physical caregiving like cleaning or helping to get dressed. Note that we used the same data and analysis in a Dutch article which has a different framing and starting point as it focuses on the effects of the COVID pandemic on the specific case of Dutch caregivers and the rules of the Dutch government (see Verbakel et al. 2021).

## Results

The descriptive statistics in Table 1 show that of the 1270 caregiving situations under research, 14% stopped, in 16% the caregiver cared less, 44% did not change, and in 16% the caregiver cared more.

**Table 1:** Descriptive statistics

		Range	Percentage	Mean	S.D
*Level 1—Caregiving situation*					
*Changes in care provision*					
Stopped care		0/1	13.8		
Cared less		0/1	25.7		
No change in care		0/1	44.3		
Cared more		0/1	16.3		
*Do I want to?* increase in worries		1–5		3.5	0.9
*Do I have to? * changes in formal care					
No change in formal care		0/1	69.8		
Reduction in formal care		0/1	20.9		
Increase in formal care	0/1		7.0		
In-and decrease in formal care		0/1	2.3		
*Can I?* feeling less restricted		1–5		3.0	1.3
*Relationship type*					
Partner		0/1	12.3		
(in-law, step-)parent		0/1	39.9		
(step-)child		0/1	9.1		
Other family members		0/1	16.9		
Friends or neighbours		0/1	21.8		
*Living situation*					
Same household		0/1	17.2		
Independent household		0/1	72.1		
Nursing home		0/1	10.7		
*Memory problems*					
No problems		0/1	51.5		
Some problems		0/1	35.0		
Serious problems		0/1	13.5		
*Mental health problems*					
No problems		0/1	62.1		
Some problems		0/1	29.5		
Serious problems		0/1	8.4		
*ADL*		0–3		0.5	0.8
*IADL*		0–3		1.2	1.2
*Caregiving intensity in March in hours*		1–140		6.1	11.1
*Level 2—Caregiver*					
*Sex*					
Men		0/1	43.7		
Women		0/1	56.3		
*Age*		16–79		55.8	14.6
*Paid work*					
Not working		0/1	51.8		
Working		0/1	48.2		
*Children*					
No children in the household in May		0/1	63.7		
Children in the household in May		0/1	36.3		
*Number of caregiving situations*		1–6		1.4	0.8

**Fig. 1:**
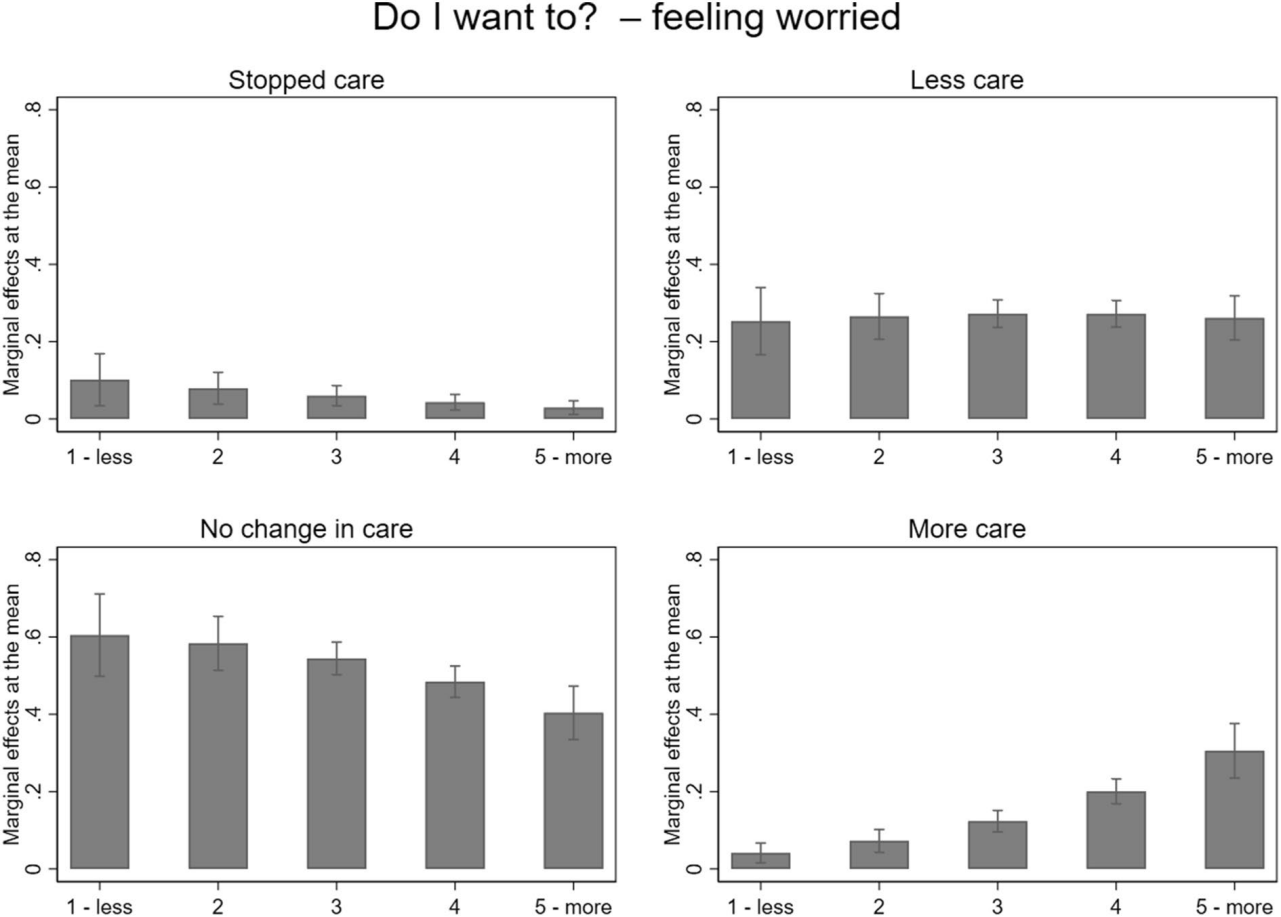
Marginal Effects at the mean of having stopped caring, cared less, no change in care, or cared more for the determinant *Do I want to?* indicated by caregivers’ worries about the care recipient during compared to before the lockdown (‘less’ indicates feeling less worried and ‘more’ indicates feeling more worried) based on the multinominal multilevel regression

Table 2 shows that, on a 5%-significance level, being more worried about the care recipient during the lockdown than before explained why a caregiver provided more care during the lockdown (MEM = 0.08). The predicted probabilities show that in caregiving situations in which the caregiver felt less worried about the care receiver during the lockdown than before, the predicted probability to care more was only 12% compared to 31% if the caregiver’s worries about the care recipient increased a lot (Fig. 1). Feeling less worried partly explained why a caregiver cared the same amount (MEM = 0.06) or stopped caregiving during the first lockdown (MEM = 0.02) (Table 2). For caring less, we did not find a statistical difference.

**Table 2:** Marginal Effects at the mean (MEM) of the multinomial multilevel regression on the three determinants of the *Informal Care Model*

Level 1*—*Caregiving situation	Stopped care		Cared less		No change in care		Cared more	
	MEM	SE	MEM	SE	MEM	SE	MEM	SE
*Do I want to?* increase in worries	− 0.02*	(0.01)	− 0.00	(0.02)	− 0.06**	(0.02)	0.08***	(0.01)
*Do I have to? * changes in formal care (ref. no change)								
reduction in formal care	− 0.01	(0.01)	− 0.03	(0.03)	− 0.08	(0.04)	0.12***	(0.04)
increase in formal care	− 0.03	(0.01)	− 0.02	(0.06)	0.03	(0.07)	0.02	(0.05)
in-and decrease in formal care	− 0.01	(0.03)	− 0.13	(0.07)	0.16	(0.11)	− 0.02	(0.08)
*Can I?* feeling less restricted	− 0.05***	(0.01)	− 0.11***	(0.01)	0.14***	(0.02)	0.02*	(0.01)
*Relationship type (ref. partner)*								
(in-law, step-)parent	0.03	(0.02)	− 0.04	(0.09)	− 0.09	(0.08)	0.10*	(0.05)
(step-)child	0.05	(0.03)	− 0.12	(0.09)	0.05	(0.09)	0.02	(0.04)
other family members	0.10**	(0.03)	− 0.04	(0.09)	− 0.11	(0.09)	0.05	(0.05)
friends or neighbours	0.06*	(0.02)	− 0.05	(0.09)	− 0.02	(0.09)	0.01	(0.05)
*Living situation (ref. independent living)*								
same household	− 0,04*	(0,02)	− 0,15**	(0,05)	0,07	(0,07)	0,12*	(0,06)
nursing home	0,11**	(0,04)	0,10	(0,06)	− 0,12	(0,07)	− 0,10***	(0,03)
*Memory problems (ref. no problems)*								
some problems	− 0,02*	(0,01)	− 0,01	(0,03)	0,01	(0,04)	0,02	(0,02)
serious problems	0,01	(0,02)	− 0,00	(0,05)	− 0,10	(0,06)	0,09	(0,05)
*Mental health problems (ref. no problems)*								
some problems	− 0,02	(0,01)	0,04	(0,03)	− 0,04	(0,04)	0,02	(0,03)
serious problems	− 0,05***	(0,01)	0,03	(0,06)	0,06	(0,06)	− 0,04	(0,04)
*ADL*	0.01	(0.01)	0.04*	(0.02)	− 0.05	(0.02)	− 0.01	(0.02)
*IADL*	− 0.00	(0.01)	− 0.03*	(0.01)	0.03	(0.02)	0.01	(0.01)
*Caregiving intensity in March (divided by 10 hours)*	− 0.06***	(0.02)	− 0.02	(0.02)	0.04*	(0.02)	0.04***	(0.01)
*Level 2—Caregiver*								
Women (ref. men)	0.05***	(0.01)	0.01	(0.03)	− 0.09**	(0.03)	0.03	(0.02)
Age	0.00	(0.00)	0.00	(0.00)	0.00	(0.00)	− 0.00	(0.00)
Working (ref. not working)	− 0.01	(0.01)	0.03	(0.03)	− 0.09*	(0.04)	0.07**	(0.02)
Children living in the household (ref. no children)	− 0.02	(0.01)	− 0.05	(0.03)	0.02	(0.04)	0.05*	(0.03)
Number of caregiving situations	0.01	(0.01)	0.01	(0.02)	− 0.02	(0.02)	0.01	(0.01)

*** *p* < 0,001; ** *p* < 0,01; * *p* < 0,05, *N* = 1270 caregiving situation of 1014 caregivers

**Fig. 2:**
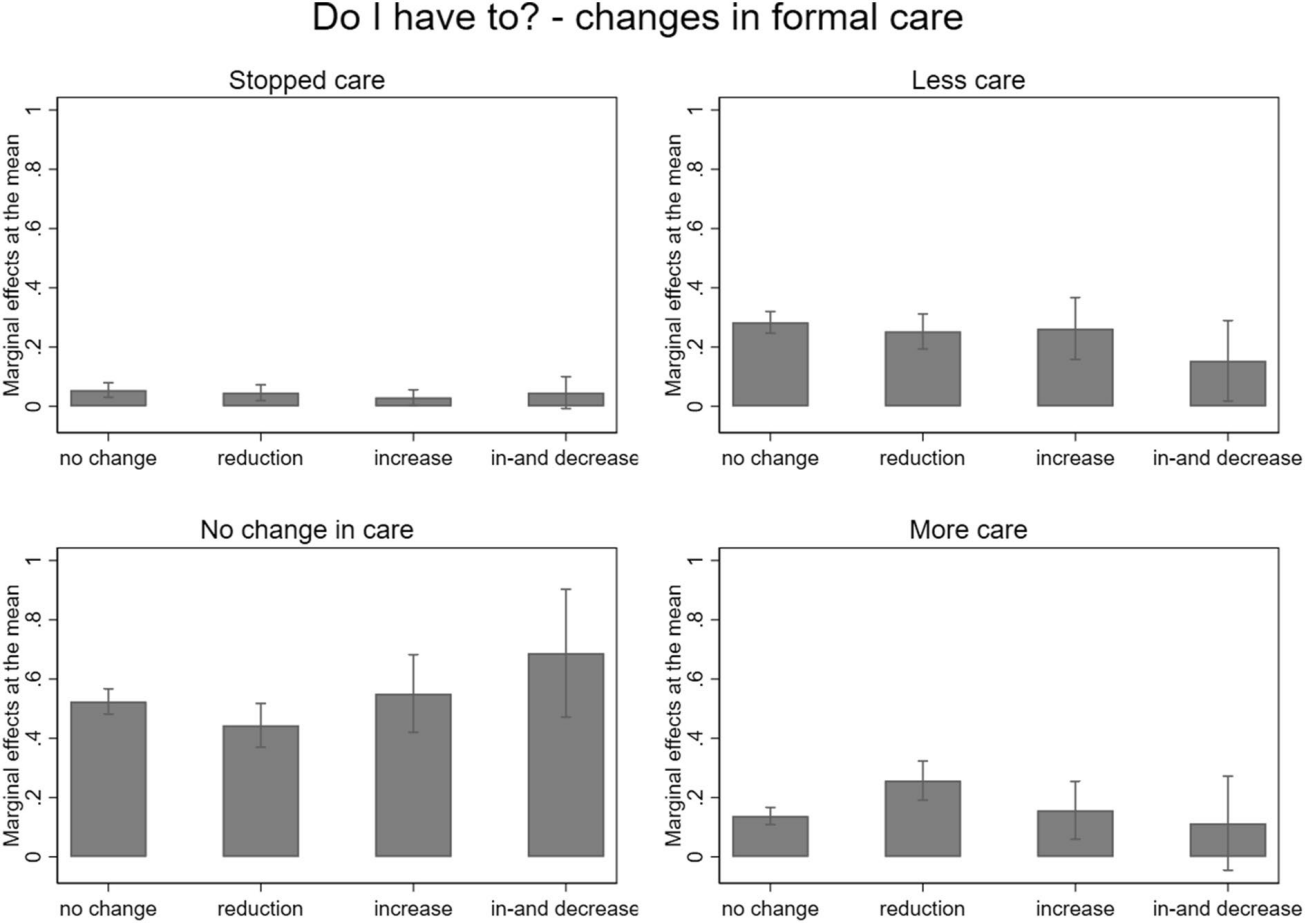
Marginal Effects at the mean of having stopped caring, cared less, no change in care, or cared more for the determinant *Do I have to?* indicated by changes in formal care provided to the care recipient during compared to before the lockdown based on the multinominal multilevel regression

There was no statistical evidence that an increase, an in-and decrease, or stable formal care provision did statistically influence changes in caregiving intensity. However, a reduction in formal care during the first lockdown was, on a 5%-significance level, related to caring more (MEM = 0.12). If formal care to a care receiver was reduced, their caregivers had a 12% higher likelihood to care more. More precisely, the predicted probabilities show that informal caregivers were 26% more likely to increase their care provision if their care receiver was confronted with reduced formal care and only 14% more likely to do so if the formal care to their care receiver had remained stable (see Fig. 2).

**Fig. 3:**
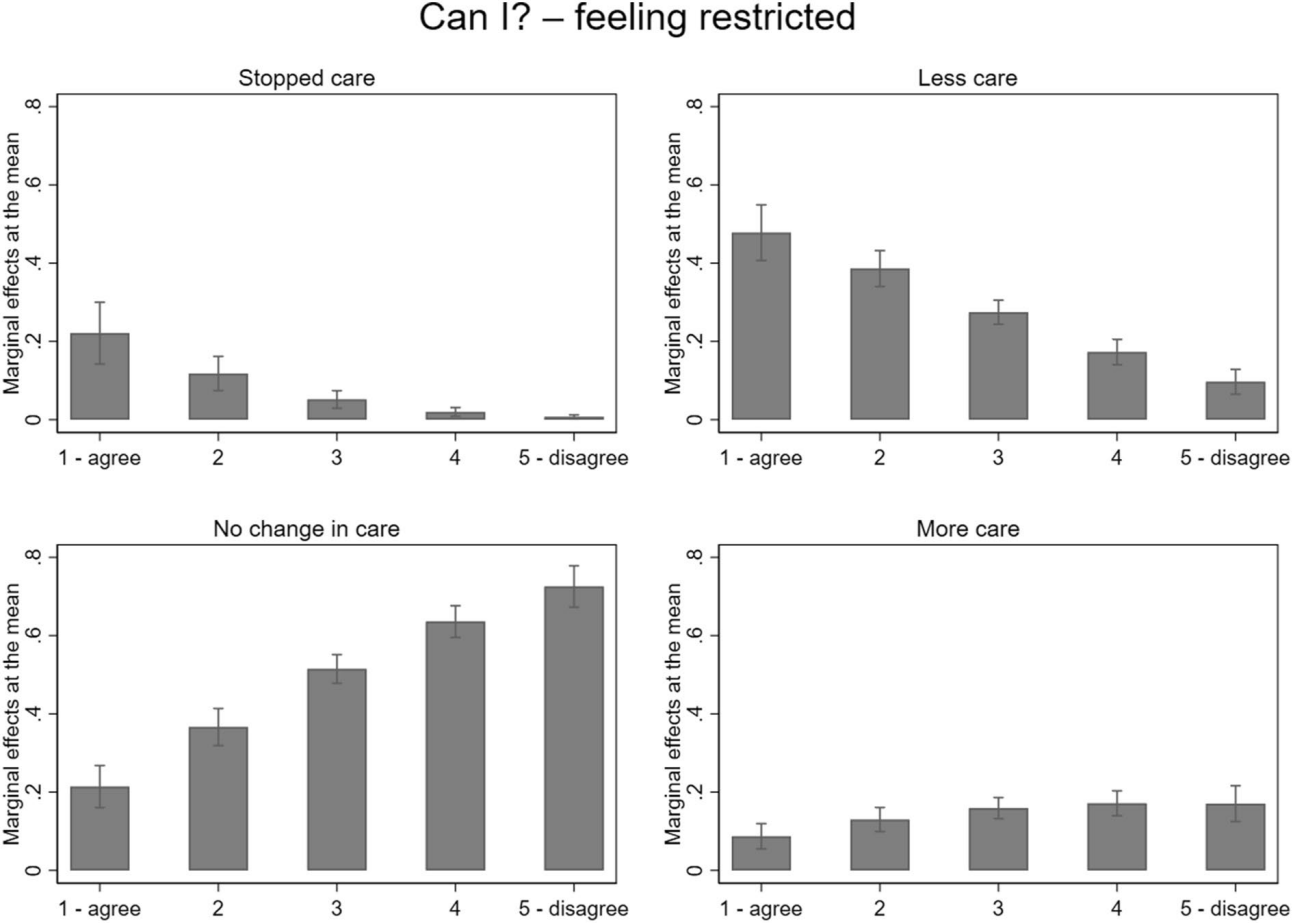
Marginal Effects at the mean of having stopped caring, cared less, no change in care, or cared more for the determinant *Can I?* indicated by caregivers’ assessment whether they felt restricted in their care provision by the measurements of the Dutch government to control the corona virus (‘agree’ indicates feeling highly restricted, and ‘disagree’ indicates not feeling restricted) based on the multinominal multilevel regression

Those who did not feel restricted by the Dutch government’s measures to control the coronavirus had a lower likelihood to stop or lower care provision but also had a higher likelihood to care the same amount or more, than those who felt strongly restricted. The effect sizes of the marginal effects can be interpreted as follows: for each additional step on the 5-point scale of the *Can I?* variable, caregivers had, in that particular caregiving situation, a 5% lower chance to have stopped, an 11% lower chance to have cared less, a 14% higher chance to have cared the same, and a 2% higher chance to have cared more. These effect sizes also show that *Can I?* was most influential for providing less care and the least influential for providing more care. For all outcome categories, we see a clear hierarchy in the predicted probabilities between low and high values on the *Can I?* variables (see Fig. 3). For instance, the predicted probability to reduce care was highest among caregivers who felt restricted the most (48%) and lowest among those who felt restricted the least (10%).

Two additional models—one that excluded situations in which care was provided to care receivers in nursing homes and one that excluded care situations in which emotional support was the only type of care provided before the lockdown—showed that, compared to the baseline analyses, the direction of the effects in the additional analyses was identical, the effect sizes were highly similar, and so were the significance levels. One relevant difference was that both additional models did not replicate that care intensity was increased in care situations in which the caregiver felt no restrictions at all; they did replicate, however, that feeling restricted reduced caregiving intensity.

## Discussion

In this paper, we used the external social shock introduced by the COVID-19 pandemic to test the *Informal Care Model* in a dynamic rather than a static design. We tested whether intrapersonal *changes* in the determinants (*Do I want to?*, *Do I have to?*, and *Can I?*) were related to *changes* in the intensity of informal caregiving. Testing existing theoretical models in new ways is valuable, as it provides insight into the scope of the theory. We used Dutch data based on a representative sample collected in March and July 2020 (1270 care situations of 1014 caregivers). Among the caregiving situations under study, 14% stopped during the Spring 2020 lockdown, in 16% the caregiver cared less, 44% did not change in intensity, and in 16% the caregiver cared more.

We found that the individual determinants derived from the *Informal Care Model* indeed helped to understand the conditions under which caregivers changed their care provision. Marginal effects showed (after correcting for series of control variables) high effect sizes. Our expectation regarding *Do I want to?* was mostly supported by our results. We observed that increased worries, indicating that caregivers wanted to care more, led to a change towards more caregiving. We found partial evidence for our expectation regarding the association between *Do I have to*? and changes in the intensity of informal care provision. The reduction in formal care, thus a higher level of having to care, was related to an increase in caregiving intensity during the lockdown, but not to caring less, a stable formal care provision and caring more.

Especially our outcomes with regard to *Can I?,* proxied by not feeling restricted by the measures of the government, were in accordance with our expectations. If caregivers felt strongly restricted in their caregiving situations by the government measures, they were more likely to stop or reduce providing care. At the same time, caregivers who did not feel restricted were more likely to increase their caregiving intensity. That we found the most support for the *Can I?* determinant is not surprising since the rules of the Dutch government to keep at least a 1.5-m distance from people, to avoid contact, and to stay home with mild symptoms practically hampered personal caregiving to a large extent. In this study, we lacked information to directly test the extent to which changes in time availability were associated with changes in caregiving intensity, despite the fact that especially work circumstances and time spent on child care were heavily affected by the pandemic. This would have been an interesting additional indicator of *Can I?*

## Conclusion

We conclude therefore that the *Informal Care Model* is a useful framework to derive hypotheses about changes in informal care provision. This study shows that the scope of the theoretical model is wider than its static starting point. Our additional models that restricted the sample of caregivers to groups that are typically used in other studies, namely those who provide informal care to community-dwelling care receivers and those who provided practical or physical help (and not only emotional support), largely confirmed the patterns we found. This shows that the *Informal Care Model* was rather insensitive to different definitions of informal caregivers. Our finding that changes in conditions explains changes in informal caregiving intensity have societal implications too. Western societies will face an increasing demand for care in the near future. Many governments, including the Dutch government, count on informal care as one way of meeting this higher demand for care. This study showed that caregivers increased their caregiving intensity when they experienced changes in the extent to which they wanted to, had to, and were able to provide care. Part of these changed conditions emerged from government policies, introduced as a response to the COVID 19 pandemic.

This study suggests that governments have options to affect the informal caregiving intensity, possibly also in times that are not dominated by COVID 19. Policies could, for instance, reduce (or increase) the availability of formal care (*Do I have to?*), take away barriers to provide care, such as leave regulations or compensation for financial losses caused by caregiving (*Can I?*), or strengthen the willingness to provide care (*Do I want to?*) by increasing the recognition of informal caregivers or by trying to influence care attitudes of citizens by pointing out—in national campaigns—the need to help each other. Important, however, is that for sustainable care provision higher intensity should not come at the cost of higher burden of specific groups of informal caregivers (De Klerk et al. 2021, see Raiber and Verbakel 2021 for gendered effects), but also not at the expense of the wellbeing of institutionalized elderly (Jeanneau et al. 2020).

This study has also limitations. Because of the setup of our data collection, in which caregivers who provided care in March 2020 were reapproached in July 2020, our sample did not include caregiving situations that started after (and perhaps due to) the lockdown. We can therefore not draw conclusions on the take-up of informal care. To increase such insight and test the *Informal Care Model* more extensively, future research should include a longitudinal design including existing and new caregiving situations. Furthermore, only caregivers aged 78 and under were examined, implying that the oldest caregivers have not been taken into account. Older caregivers may have reacted differently to the lockdown and associated measures. This information is missing from the LISS panel. Further research will have to determine if the *Informal Care Model* is also suitable for older caregivers.

All in all, did the external shock by the COVID-19 pandemic provide a good framework to apply and empirically test the *Informal Care Model*. Our assessment showed that the model is easily applicable, usable in different populations and finds empirical support, and is thus useful as an analytical tool to look at the increasing care provision that is expected to be needed in the future. Moreover, in efforts of European governments to prepare for a stable informal care provision in countries, policymakers may pay attention to the principles of the Informal Care Model and its empirical tests, as it provides a useful policy tool for realistic assumptions about behavioural responses of informal caregivers to policy measures.

## Data Availability

The first part of the data “Retrospective informal care career: Main measurement” that support the findings of this study is openly available in the LISS data archive, CentERdata at 10.17026/dans-xyf-v7vu. The second part of the data “Retrospective informal care career: Follow-Up measurement” that support the findings of this study similarly is openly available in the LISS data archive, CentERdata at 10.17026/dans-z6w-rd24.

## Appendix

See Tables

**Table 3:** Marginal Effects at the mean (MEM) of the multinomial multilevel regression on the three determinants of the *Informal Care Model* when excluding care recipients living in nursing homes

	Stopped care		Cared less		No change in care		Cared more	
Level 1*—*caregiving situation	MEM	SE	MEM	SE	MEM	SE	MEM	SE
*Do I want to?* increase in worries	-0.01*	(0.01)	-0.01	(0.02)	-0.07**	(0.02)	0.09***	(0.02)
Do I have to? changes in formal care (ref. no change)								
reduction in formal care	-0.00	(0.01)	-0.01	(0.04)	-0.09*	(0.05)	0.10**	(0.04)
increase in formal care	-0.03*	(0.01)	-0.04	(0.07)	0.01	(0.09)	0.06	(0.08)
*Can I?* feeling less restricted	-0.04***	(0.01)	-0.11***	(0.01)	0.13***	(0.02)	0.02	(0.01)
*Relationship type (ref. partner)*								
(in-law, step-)parent	0.02	(0.02)	-0.04	(0.08)	-0.11	(0.08)	0.13**	(0.04)
(step-)child	0.03	(0.02)	-0.11	(0.09)	0.05	(0.09)	0.03	(0.04)
other family members	0.08*	(0.03)	-0.02	(0.09)	-0.13	(0.09)	0.07	(0.06)
friends or neighbours	0.05*	(0.02)	-0.05	(0.08)	-0.03	(0.08)	0.03	(0.05)
*Living situation (ref. independent living)*								
same household	-0.03*	(0.02)	-0.15**	(0.05)	0.05	(0.07)	0.13*	(0.06)
*Memory problems (ref. no problems)*								
some problems	-0.02	(0.01)	0.02	(0.03)	-0.02	(0.04)	0.02	(0.03)
serious problems	0.00	(0.02)	0.02	(0.06)	-0.11	(0.07)	0.09	(0.06)
*Mental health problems (ref. no problems)*								
some problems	-0.02	(0.01)	0.04	(0.03)	-0.05	(0.04)	0.03	(0.03)
serious problems	-0.03	(0.02)	0.03	(0.07)	0.07	(0.07)	-0.07	(0.04)
*ADL*	0.01	(0.01)	0.03	(0.02)	-0.04	(0.03)	-0.01	(0.02)
*IADL*	-0.01	(0.01)	-0.03*	(0.02)	0.03	(0.02)	0.01	(0.01)
*Caregiving intensity in March (divided by 10 h)*	-0.04**	(0.01)	-0.03	(0.02)	0.03	(0.02)	0.04***	(0.01)
*Level 2—Caregiver*								
Women (ref. men)	0.04*	(0.01)	0.04	(0.03)	-0.11**	(0.04)	0.04	(0.03)
Age	0.00	(0.00)	0.00	(0.00)	-0.00	(0.00)	-0.00	(0.00)
Working (ref. not working)	0.04*	(0.01)	0.04	(0.03)	-0.11**	(0.04)	0.04	(0.03)
Children living in the household (ref. none)	-0.02	(0.01)	-0.04	(0.03)	0.00	(0.04)	0.06*	(0.03)
Number of caregiving situations	0.01	(0.01)	0.01	(0.02)	-0.03	(0.02)	0.01	(0.01)

*** *p* < 0,001; ** *p* < 0,01; * *p* < 0,05, *N* = 1121 caregiving situation of 892 caregivers*. Note:* In-and decrease in formal care had too little cases to remain included

3 and

**Table 4:** Marginal Effects at the mean (MEM) of the multinomial multilevel regression on the three determinants of the *Informal Care Model* when excluding care situations where only emotional support was given

	Stopped care		Cared less			No change in care		Cared more	
Level 1*—*Caregiving situation	MEM	SE		MEM	SE	MEM	SE	MEM	SE
*Do I want to?* increase in worries	-0.01	(0.01)		-0.00	(0.02)	-0.08***	(0.02)	0.09***	(0.02)
*Do I have to?* changes in formal care (ref. no change)									
reduction in formal care	-0.02	(0.01)		-0.01	(0.03)	-0.10*	(0.05)	0.12**	(0.04)
increase in formal care	-0.03	(0.01)		0.02	(0.06)	-0.00	(0.07)	0.01	(0.06)
in-and decrease in formal care	-0.02	(0.02)		-0.09	(0.08)	0.11	(0.12)	-0.01	(0.10)
*Can I?* feeling less restricted	-0.04**	(0.01)		-0.10***	(0.02)	0.12***	(0.02)	0.01	(0.01)
*Relationship type (ref. partner)*									
(in-law, step- parent	0.02	(0.03)		-0.06	(0.08)	-0.07	(0.08)	0.11*	(0.04)
(step-)child	0.03	(0.03)		-0.12	(0.09)	0.06	(0.09)	0.03	(0.05)
other family members	0.06	(0.03)		-0.05	(0.08)	-0.08	(0.09)	0.07	(0.06)
friends or neighbours	0.03	(0.03)		-0.07	(0.07)	-0.00	(0.08)	0.05	(0.05)
*Living situation (ref. independent living)*									
same household	-0.03*	(0.01)		-0.16***	(0.05)	0.06	(0.07)	0.14*	(0.07)
nursing home	0.11*	(0.05)		0.10	(0.06)	-0.10	(0.07)	-0.11**	(0.04)
*Memory problems (ref. no problems)*									
some problems	-0.02	(0.01)		-0.04	(0.03)	0.04	(0.04)	0.02	(0.03)
serious problems	-0.00	(0.02)		-0.04	(0.05)	-0.08	(0.07)	0.12*	(0.06)
*Mental health problems (ref. no problems)*									
some problems	-0.01	(0.01)		0.06	(0.03)	-0.07	(0.04)	0.02	(0.03)
serious problems	-0.04*	(0.02)		0.07	(0.06)	0.06	(0.07)	-0.09*	(0.04)
*ADL*	0.01	(0.01)		0.05*	(0.02)	-0.05	(0.02)	-0.01	(0.02)
*IADL*	-0.01	(0.01)		-0.03*	(0.01)	0.02	(0.02)	0.01	(0.01)
*Caregiving intensity in March (divided by 10 h)*	-0.04*	(0.01)		-0.04	(0.02)	0.03	(0.02)	0.04***	(0.01)
*Level 2—Caregiver*									
Women (ref. men)	0.03*	(0.01)		0.01	(0.03)	-0.07	(0.03)	0.03	(0.03)
Age	0.00	(0.00)		-0.00	(0.00)	0.00	(0.00)	-0.00	(0.00)
Working (ref. not working)	-0.01	(0.01)		0.03	(0.03)	-0.09*	(0.04)	0.06*	(0.03)
Children living in the household (ref. none)	-0.01	(0.01)		-0.07	(0.03)	0.02	(0.04)	0.06*	(0.03)
Number of caregiving situations	0.01	(0.01)		0.01	(0.02)	-0.03	(0.02)	0.01	(0.01)

****p* < 0,001; ** *p* < 0,01; * *p* < 0,05, *N* = 1092 caregiving situation of 883 caregivers

4.

## Footnotes

1 Raiber et al. (2021) found that the COVID-19 lockdown, which started the March 15, did not influence the response for the March data by checking if answer patterns of respondents filling in the survey before or after the lockdown was different.

2 The respondents did get the following definition for informal care: “Below we will ask you to indicate all the people known to you to whom you have ever offered informal care on account of their health issues. These people could be your partner, a family member, a friend, neighbor, acquaintance or colleague who needs or needed help because of physical, psychological or mental limitations or because of old age. Examples of informal care are doing household chores, helping with washing and dressing, keeping company, providing transport or performing odd jobs. You may have done so for a short period or for a long period. It could involve people known to you to whom you provided care in the past, but could also involve people known to you to whom are providing care at present. Care provided as part of your occupation or as a volunteer does not count. Please take a moment to think about the people known to you to whom you have provided or are providing informal care on account of health issues.”(Verbakel and CentErdata 2021). The care recipients can live together with the caregiver, in an independent household, or in a care institution (this is later controlled for in our models).

3 For the variables working and children, we added monthly LISS panel data from May 2020.
